# 2,3-Dibromo-3-(4-bromo­phen­yl)-1-[3-(4-meth­oxy­phen­yl)sydnon-4-yl]propan-1-one

**DOI:** 10.1107/S1600536811010002

**Published:** 2011-03-23

**Authors:** Hoong-Kun Fun, Madhukar Hemamalini, Nithinchandra, Balakrishna Kalluraya

**Affiliations:** a X-ray Crystallography Unit, School of Physics, Universiti Sains Malaysia, 11800 USM, Penang, Malaysia; b Department of Studies in Chemistry, Mangalore University, Mangalagangotri, Mangalore 574 199, India

## Abstract

In the title compound {systematic name: 4-[2,3-dibromo-3-(4-bromo­phen­yl)propano­yl]-3-(4-meth­oxy­phen­yl)-1,2,3-oxa­dia­zol-3-ylium-5-olate}, C_18_H_13_Br_3_N_2_O_4_, the central oxadiazole ring, which is essentially planar with a maximum deviation of 0.016 (3) Å, makes dihedral angles of 29.98 (16) and 52.04 (16)°, respectively, with the terminal bromo-substituted and meth­oxy-substituted benzene rings. An intra­molecular C—H⋯O hydrogen bond generates an *S*(6) ring motif.

## Related literature

For applications of sydnones, see: Rai *et al.* (2008 ▶); Jyothi *et al.* (2008 ▶). For details of chalcones, see: Rai *et al.* (2007 ▶). For graph-set notation, see: Bernstein *et al.* (1995 ▶). 
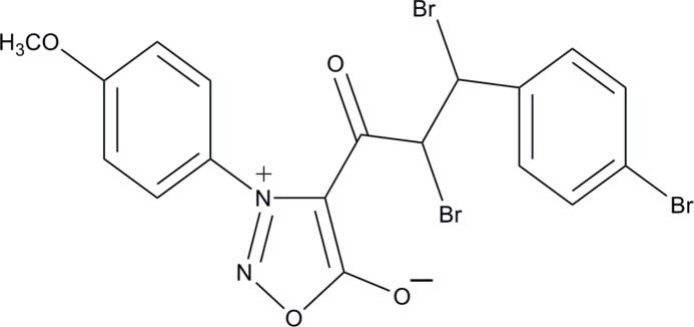


## Experimental

### 

#### Crystal data

C_18_H_13_Br_3_N_2_O_4_
*M* *_r_* = 561.03
Monoclinic, 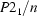

*a* = 7.8024 (1) Å
*b* = 24.0261 (3) Å
*c* = 10.8211 (1) Å
β = 108.848 (1)°
*V* = 1919.76 (4) Å^3^
*Z* = 4
Mo *K*α radiation
μ = 6.33 mm^−1^
*T* = 296 K
0.39 × 0.27 × 0.13 mm

#### Data collection

Bruker SMART APEXII CCD area-detector diffractometer
Absorption correction: multi-scan (*SADABS*; Bruker, 2009) ▶ *T*_min_ = 0.191, *T*_max_ = 0.496
20615 measured reflections
5841 independent reflections
3490 reflections with *I* > 2σ(*I*)
*R*_int_ = 0.034

#### Refinement

*R*[*F* ^2^ > 2σ(*F* ^2^)] = 0.041
*wR*(*F* ^2^) = 0.101
*S* = 1.00
5841 reflections
245 parameters
H-atom parameters constrained
Δρ_max_ = 0.63 e Å^−3^
Δρ_min_ = −0.74 e Å^−3^

### 

Data collection: *APEX2* (Bruker, 2009 ▶); cell refinement: *SAINT* (Bruker, 2009 ▶); data reduction: *SAINT*; program(s) used to solve structure: *SHELXTL* (Sheldrick, 2008 ▶); program(s) used to refine structure: *SHELXTL*; molecular graphics: *SHELXTL*; software used to prepare material for publication: *SHELXTL* and *PLATON* (Spek, 2009 ▶).

## Supplementary Material

Crystal structure: contains datablocks global, I. DOI: 10.1107/S1600536811010002/is2691sup1.cif

Structure factors: contains datablocks I. DOI: 10.1107/S1600536811010002/is2691Isup2.hkl

Additional supplementary materials:  crystallographic information; 3D view; checkCIF report

## Figures and Tables

**Table 1:** Hydrogen-bond geometry (Å, °)

*D*—H⋯*A*	*D*—H	H⋯*A*	*D*⋯*A*	*D*—H⋯*A*
C8—H8*A*⋯O2	0.98	2.35	3.032 (4)	126

**Fig. 1.**
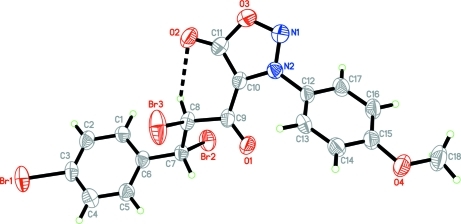
The asymmetric unit of the title compound, showing 50% probability displacement ellipsoids and the atom-numbering scheme. The intramolecular hydrogen bond is shown as a dashed line.

**Fig. 2.**
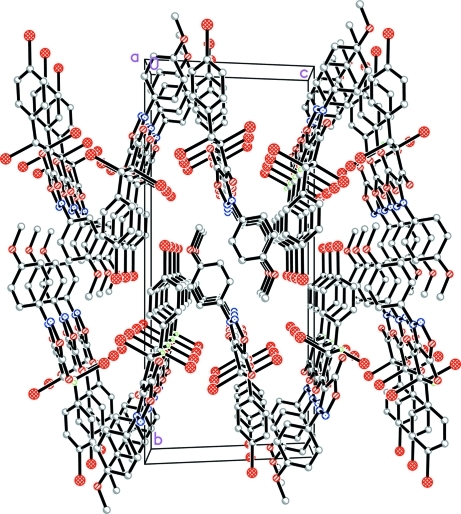
The crystal packing of the title compound (I).

## supplementary crystallographic information

### Comment

Sydnones constitute a well-defined class of mesoionic compounds that contain the 1,2,3-oxadiazole ring system. The study of sydnones still remains a field of interest because of their electronic structure and also because of the varied types of biological activities (Rai *et al.*, 2008). Recently, sydnone derivatives were found to exhibit promising antimicrobial properties (Jyothi *et al.*, 2008). Chalcones were obtained by the base-catalyzed condensation of 4-acetyl-3-aryl sydnones with aromatic aldehydes in alcoholic medium employing sodium hydroxide as catalyst at 0–5 °C. Bromination of chalcones with bromine in glacial acetic acid afforded dibromo chalcones (Rai *et al.*, 2007).

The molecular structure of the title compound is shown in Fig. 1. The oxadiazole (N1/N2/O3/C10/C11) ring is essentially planar, with a maximum deviation of 0.016 (3) Å for atom C11.  The central oxadiazole ring makes dihedral angles of  29.98 (16)°  and 52.04 (16)°, with the terminal bromo-substituted phenyl (C1–C6) and the methoxy-substituted phenyl( C12–C17) rings, respectively.

In the crystal, (Fig. 2), there is an intramolecular C8—H8A···O2 (Table 1) hydrogen bond, which generates an *S*(6) ring motif (Bernstein *et al.*, 1995).

### Experimental

1-(3^1^-Phenylsydnon-4^1^-yl)-3-(*p*-bromophenyl)- propen-1-one (0.01 mol) was dissolved in glacial acetic acid (25–30 ml) by gentle warming. A solution of bromine in glacial acetic acid (30% w/v) was added to it with constant stirring till the yellow colour of the bromine persisted. The reaction mixture was stirred at room temperature for 1–2 hours. The solid which separated was filtered, washed with methanol and dried. It was then recrystallized from ethanol. Crystals suitable for X-ray analysis were obtained from 1:2 mixtures of DMF and ethanol by slow evaporation.

### Refinement

All H atoms were positioned geometrically (C—H = 0.93–0.98 Å) and were refined  using a riding model, with *U*_iso_(H) = 1.2 or 1.5*U*_eq_(C). A rotating group model was used for the methyl group.

### Crystal data

**Table:**

C_18_H_13_Br_3_N_2_O_4_	*F*(000) = 1088
*M**_r_* = 561.03	*D*_x_ = 1.941 Mg m^−^^3^
Monoclinic, *P*2_1_/*n*	Mo *K*α radiation, λ = 0.71073 Å
Hall symbol: -P 2yn	Cell parameters from 6367 reflections
*a* = 7.8024 (1) Å	θ = 2.6–28.3°
*b* = 24.0261 (3) Å	µ = 6.33 mm^−^^1^
*c* = 10.8211 (1) Å	*T* = 296 K
β = 108.848 (1)°	Plate, colourless
*V* = 1919.76 (4) Å^3^	0.39 × 0.27 × 0.13 mm
*Z* = 4	

### Data collection

**Table:**

Bruker SMART APEXII CCD area-detector diffractometer	5841 independent reflections
Radiation source: fine-focus sealed tube	3490 reflections with *I* > 2σ(*I*)
graphite	*R*_int_ = 0.034
φ and ω scans	θ_max_ = 30.5°, θ_min_ = 1.7°
Absorption correction: multi-scan (*SADABS*; Bruker, 2009)	*h* = −11→11
*T*_min_ = 0.191, *T*_max_ = 0.496	*k* = −34→23
20615 measured reflections	*l* = −14→15

### Refinement

**Table:**

Refinement on *F*^2^	Primary atom site location: structure-invariant direct methods
Least-squares matrix: full	Secondary atom site location: difference Fourier map
*R*[*F*^2^ > 2σ(*F*^2^)] = 0.041	Hydrogen site location: inferred from neighbouring sites
*wR*(*F*^2^) = 0.101	H-atom parameters constrained
*S* = 1.00	*w* = 1/[σ^2^(*F*_o_^2^) + (0.0481*P*)^2^ + 0.1633*P*] where *P* = (*F*_o_^2^ + 2*F*_c_^2^)/3
5841 reflections	(Δ/σ)_max_ = 0.001
245 parameters	Δρ_max_ = 0.63 e Å^−^^3^
0 restraints	Δρ_min_ = −0.74 e Å^−^^3^

### Special details

**Table:**

Geometry. All s.u.'s (except the s.u. in the dihedral angle between two l.s. planes) are estimated using the full covariance matrix. The cell s.u.'s are taken into account individually in the estimation of s.u.'s in distances, angles and torsion angles; correlations between s.u.'s in cell parameters are only used when they are defined by crystal symmetry. An approximate (isotropic) treatment of cell s.u.'s is used for estimating s.u.'s involving l.s. planes.
Refinement. Refinement of F^2^ against ALL reflections. The weighted R-factor wR and goodness of fit S are based on F^2^, conventional R-factors R are based on F, with F set to zero for negative F^2^. The threshold expression of F^2^ > 2σ(F^2^) is used only for calculating R-factors(gt) etc. and is not relevant to the choice of reflections for refinement. R-factors based on F^2^ are statistically about twice as large as those based on F, and R- factors based on ALL data will be even larger.

### Fractional atomic coordinates and isotropic or equivalent isotropic displacement parameters (Å^2^)

**Table:**

	*x*	*y*	*z*	*U*_iso_*/*U*_eq_	
Br1	1.14574 (6)	−0.038999 (13)	0.37333 (4)	0.06333 (13)	
Br2	0.95173 (5)	0.252012 (13)	0.22636 (3)	0.05191 (11)	
Br3	0.87928 (7)	0.183591 (15)	0.60488 (4)	0.07974 (16)	
O1	0.9670 (3)	0.31433 (8)	0.5109 (2)	0.0525 (6)	
O2	0.4433 (3)	0.22666 (9)	0.3829 (2)	0.0557 (6)	
O3	0.3565 (3)	0.31283 (9)	0.4242 (2)	0.0513 (5)	
O4	1.0534 (3)	0.52989 (8)	0.7386 (2)	0.0541 (6)	
N1	0.4365 (3)	0.36133 (10)	0.4786 (2)	0.0462 (6)	
N2	0.6087 (3)	0.35365 (9)	0.5050 (2)	0.0347 (5)	
C1	0.9177 (4)	0.11794 (11)	0.2936 (3)	0.0427 (7)	
H1A	0.8111	0.1332	0.2379	0.051*	
C2	0.9488 (4)	0.06109 (12)	0.2902 (3)	0.0468 (7)	
H2A	0.8635	0.0382	0.2329	0.056*	
C3	1.1067 (4)	0.03920 (11)	0.3725 (3)	0.0420 (7)	
C4	1.2369 (4)	0.07204 (13)	0.4552 (3)	0.0479 (8)	
H4A	1.3447	0.0565	0.5084	0.057*	
C5	1.2064 (4)	0.12855 (12)	0.4587 (3)	0.0420 (7)	
H5A	1.2945	0.1512	0.5145	0.050*	
C6	1.0445 (4)	0.15199 (11)	0.3793 (3)	0.0362 (6)	
C7	1.0097 (4)	0.21271 (11)	0.3953 (3)	0.0337 (6)	
H7A	1.1185	0.2293	0.4570	0.040*	
C8	0.8505 (4)	0.22382 (11)	0.4423 (3)	0.0395 (6)	
H8A	0.7381	0.2121	0.3755	0.047*	
C9	0.8350 (4)	0.28446 (11)	0.4789 (3)	0.0374 (6)	
C10	0.6552 (4)	0.30218 (11)	0.4736 (3)	0.0354 (6)	
C11	0.4882 (4)	0.27294 (12)	0.4223 (3)	0.0429 (7)	
C12	0.7239 (4)	0.40007 (11)	0.5653 (3)	0.0342 (6)	
C13	0.8625 (4)	0.39242 (11)	0.6808 (3)	0.0389 (7)	
H13A	0.8838	0.3576	0.7206	0.047*	
C14	0.9684 (4)	0.43718 (12)	0.7358 (3)	0.0423 (7)	
H14A	1.0633	0.4328	0.8135	0.051*	
C15	0.9355 (4)	0.48902 (11)	0.6766 (3)	0.0406 (7)	
C16	0.7926 (4)	0.49630 (12)	0.5621 (3)	0.0447 (7)	
H16A	0.7685	0.5312	0.5233	0.054*	
C17	0.6866 (4)	0.45139 (12)	0.5062 (3)	0.0433 (7)	
H17A	0.5905	0.4557	0.4291	0.052*	
C18	1.0212 (5)	0.58493 (13)	0.6871 (4)	0.0671 (10)	
H18A	1.1193	0.6087	0.7345	0.101*	
H18B	0.9100	0.5987	0.6954	0.101*	
H18C	1.0126	0.5844	0.5966	0.101*	

### Atomic displacement parameters (Å^2^)

**Table:**

	*U*^11^	*U*^22^	*U*^33^	*U*^12^	*U*^13^	*U*^23^
Br1	0.0987 (3)	0.02994 (17)	0.0680 (2)	0.00875 (16)	0.0361 (2)	0.00131 (14)
Br2	0.0698 (2)	0.04374 (19)	0.04443 (19)	0.00350 (15)	0.02161 (16)	0.00642 (13)
Br3	0.1518 (4)	0.0394 (2)	0.0769 (3)	0.0121 (2)	0.0770 (3)	0.01108 (17)
O1	0.0444 (12)	0.0336 (11)	0.0857 (16)	−0.0076 (9)	0.0296 (11)	−0.0153 (11)
O2	0.0558 (13)	0.0399 (12)	0.0737 (15)	−0.0161 (11)	0.0243 (11)	−0.0118 (11)
O3	0.0404 (12)	0.0495 (13)	0.0626 (14)	−0.0055 (10)	0.0149 (10)	−0.0070 (11)
O4	0.0582 (14)	0.0369 (12)	0.0598 (14)	−0.0091 (10)	0.0087 (11)	−0.0069 (10)
N1	0.0429 (15)	0.0402 (14)	0.0546 (15)	−0.0011 (11)	0.0145 (12)	−0.0050 (12)
N2	0.0362 (13)	0.0325 (12)	0.0370 (12)	0.0008 (10)	0.0140 (10)	−0.0008 (9)
C1	0.0403 (16)	0.0350 (15)	0.0506 (17)	0.0012 (12)	0.0116 (14)	−0.0072 (13)
C2	0.0555 (19)	0.0331 (15)	0.0527 (18)	−0.0051 (14)	0.0187 (15)	−0.0107 (14)
C3	0.0583 (19)	0.0270 (14)	0.0501 (17)	0.0016 (13)	0.0308 (15)	0.0008 (12)
C4	0.0545 (19)	0.0405 (17)	0.0468 (18)	0.0080 (15)	0.0138 (15)	0.0036 (14)
C5	0.0470 (17)	0.0357 (15)	0.0404 (16)	−0.0007 (13)	0.0102 (14)	−0.0026 (12)
C6	0.0432 (16)	0.0293 (14)	0.0398 (15)	−0.0002 (12)	0.0186 (13)	−0.0050 (11)
C7	0.0377 (15)	0.0291 (13)	0.0360 (14)	−0.0008 (11)	0.0142 (12)	−0.0021 (11)
C8	0.0493 (17)	0.0288 (14)	0.0441 (16)	−0.0053 (12)	0.0202 (13)	−0.0030 (12)
C9	0.0457 (16)	0.0268 (13)	0.0453 (16)	−0.0017 (12)	0.0225 (13)	−0.0012 (12)
C10	0.0413 (16)	0.0286 (14)	0.0397 (15)	−0.0034 (11)	0.0177 (12)	−0.0007 (11)
C11	0.0458 (17)	0.0410 (17)	0.0443 (17)	−0.0078 (14)	0.0178 (14)	0.0008 (13)
C12	0.0389 (15)	0.0261 (13)	0.0404 (15)	0.0007 (11)	0.0166 (12)	−0.0047 (11)
C13	0.0467 (17)	0.0294 (14)	0.0410 (16)	0.0092 (12)	0.0147 (13)	0.0059 (12)
C14	0.0428 (17)	0.0390 (16)	0.0407 (16)	0.0061 (13)	0.0074 (13)	−0.0013 (13)
C15	0.0457 (17)	0.0314 (15)	0.0461 (17)	0.0012 (12)	0.0168 (14)	−0.0043 (12)
C16	0.0534 (18)	0.0292 (15)	0.0476 (17)	0.0017 (13)	0.0110 (15)	0.0037 (13)
C17	0.0453 (17)	0.0361 (16)	0.0430 (16)	0.0056 (13)	0.0067 (13)	0.0032 (12)
C18	0.082 (3)	0.0320 (18)	0.084 (3)	−0.0109 (17)	0.022 (2)	−0.0017 (17)

### Geometric parameters (Å, °)

**Table:**

Br1—C3	1.903 (3)	C5—H5A	0.9300
Br2—C7	1.976 (3)	C6—C7	1.504 (4)
Br3—C8	1.956 (3)	C7—C8	1.510 (4)
O1—C9	1.211 (3)	C7—H7A	0.9800
O2—C11	1.202 (3)	C8—C9	1.525 (4)
O3—N1	1.362 (3)	C8—H8A	0.9800
O3—C11	1.411 (4)	C9—C10	1.449 (4)
O4—C15	1.364 (3)	C10—C11	1.425 (4)
O4—C18	1.426 (4)	C12—C13	1.376 (4)
N1—N2	1.293 (3)	C12—C17	1.376 (4)
N2—C10	1.363 (3)	C13—C14	1.369 (4)
N2—C12	1.449 (3)	C13—H13A	0.9300
C1—C6	1.384 (4)	C14—C15	1.386 (4)
C1—C2	1.390 (4)	C14—H14A	0.9300
C1—H1A	0.9300	C15—C16	1.385 (4)
C2—C3	1.371 (4)	C16—C17	1.375 (4)
C2—H2A	0.9300	C16—H16A	0.9300
C3—C4	1.367 (4)	C17—H17A	0.9300
C4—C5	1.381 (4)	C18—H18A	0.9600
C4—H4A	0.9300	C18—H18B	0.9600
C5—C6	1.396 (4)	C18—H18C	0.9600
			
N1—O3—C11	110.7 (2)	Br3—C8—H8A	109.8
C15—O4—C18	118.0 (2)	O1—C9—C10	124.1 (2)
N2—N1—O3	105.8 (2)	O1—C9—C8	120.6 (3)
N1—N2—C10	114.6 (2)	C10—C9—C8	115.3 (2)
N1—N2—C12	116.0 (2)	N2—C10—C11	105.1 (2)
C10—N2—C12	129.4 (2)	N2—C10—C9	126.1 (2)
C6—C1—C2	120.3 (3)	C11—C10—C9	128.5 (3)
C6—C1—H1A	119.8	O2—C11—O3	120.2 (3)
C2—C1—H1A	119.8	O2—C11—C10	136.0 (3)
C3—C2—C1	119.2 (3)	O3—C11—C10	103.8 (2)
C3—C2—H2A	120.4	C13—C12—C17	121.9 (3)
C1—C2—H2A	120.4	C13—C12—N2	119.8 (2)
C4—C3—C2	121.7 (3)	C17—C12—N2	118.2 (2)
C4—C3—Br1	118.9 (2)	C14—C13—C12	118.5 (3)
C2—C3—Br1	119.4 (2)	C14—C13—H13A	120.8
C3—C4—C5	119.2 (3)	C12—C13—H13A	120.8
C3—C4—H4A	120.4	C13—C14—C15	120.6 (3)
C5—C4—H4A	120.4	C13—C14—H14A	119.7
C4—C5—C6	120.6 (3)	C15—C14—H14A	119.7
C4—C5—H5A	119.7	O4—C15—C16	124.7 (3)
C6—C5—H5A	119.7	O4—C15—C14	115.1 (3)
C1—C6—C5	118.9 (3)	C16—C15—C14	120.2 (3)
C1—C6—C7	122.2 (2)	C17—C16—C15	119.4 (3)
C5—C6—C7	118.8 (2)	C17—C16—H16A	120.3
C6—C7—C8	114.1 (2)	C15—C16—H16A	120.3
C6—C7—Br2	110.67 (18)	C16—C17—C12	119.5 (3)
C8—C7—Br2	105.06 (18)	C16—C17—H17A	120.3
C6—C7—H7A	108.9	C12—C17—H17A	120.3
C8—C7—H7A	108.9	O4—C18—H18A	109.5
Br2—C7—H7A	108.9	O4—C18—H18B	109.5
C7—C8—C9	113.5 (2)	H18A—C18—H18B	109.5
C7—C8—Br3	110.34 (19)	O4—C18—H18C	109.5
C9—C8—Br3	103.54 (18)	H18A—C18—H18C	109.5
C7—C8—H8A	109.8	H18B—C18—H18C	109.5
C9—C8—H8A	109.8		
			
C11—O3—N1—N2	2.3 (3)	C12—N2—C10—C9	−8.6 (4)
O3—N1—N2—C10	−0.6 (3)	O1—C9—C10—N2	−1.1 (5)
O3—N1—N2—C12	−179.6 (2)	C8—C9—C10—N2	178.2 (2)
C6—C1—C2—C3	−0.2 (5)	O1—C9—C10—C11	171.4 (3)
C1—C2—C3—C4	−1.9 (5)	C8—C9—C10—C11	−9.3 (4)
C1—C2—C3—Br1	177.5 (2)	N1—O3—C11—O2	176.8 (3)
C2—C3—C4—C5	1.9 (5)	N1—O3—C11—C10	−3.0 (3)
Br1—C3—C4—C5	−177.5 (2)	N2—C10—C11—O2	−177.3 (3)
C3—C4—C5—C6	0.2 (5)	C9—C10—C11—O2	9.0 (6)
C2—C1—C6—C5	2.3 (5)	N2—C10—C11—O3	2.5 (3)
C2—C1—C6—C7	−174.2 (3)	C9—C10—C11—O3	−171.2 (3)
C4—C5—C6—C1	−2.3 (5)	N1—N2—C12—C13	125.9 (3)
C4—C5—C6—C7	174.3 (3)	C10—N2—C12—C13	−52.8 (4)
C1—C6—C7—C8	60.9 (4)	N1—N2—C12—C17	−52.1 (3)
C5—C6—C7—C8	−115.6 (3)	C10—N2—C12—C17	129.2 (3)
C1—C6—C7—Br2	−57.4 (3)	C17—C12—C13—C14	−1.7 (4)
C5—C6—C7—Br2	126.1 (2)	N2—C12—C13—C14	−179.6 (3)
C6—C7—C8—C9	170.7 (2)	C12—C13—C14—C15	0.4 (4)
Br2—C7—C8—C9	−67.9 (2)	C18—O4—C15—C16	4.2 (5)
C6—C7—C8—Br3	55.0 (3)	C18—O4—C15—C14	−176.5 (3)
Br2—C7—C8—Br3	176.43 (11)	C13—C14—C15—O4	−178.2 (3)
C7—C8—C9—O1	−24.4 (4)	C13—C14—C15—C16	1.2 (5)
Br3—C8—C9—O1	95.3 (3)	O4—C15—C16—C17	177.7 (3)
C7—C8—C9—C10	156.2 (2)	C14—C15—C16—C17	−1.6 (5)
Br3—C8—C9—C10	−84.1 (2)	C15—C16—C17—C12	0.3 (5)
N1—N2—C10—C11	−1.3 (3)	C13—C12—C17—C16	1.4 (5)
C12—N2—C10—C11	177.5 (2)	N2—C12—C17—C16	179.3 (3)
N1—N2—C10—C9	172.7 (3)		

### Hydrogen-bond geometry (Å, °)

**Table:**

*D*—H···*A*	*D*—H	H···*A*	*D*···*A*	*D*—H···*A*
C8—H8A···O2	0.98	2.35	3.032 (4)	126
